# Determinants of stunting among children aged 6 to 59 months in pastoral community, Afar region, North East Ethiopia: unmatched case control study

**DOI:** 10.1186/s40795-020-00332-z

**Published:** 2020-02-17

**Authors:** Molla Kahssay, Etsay Woldu, Abel Gebre, Surender Reddy

**Affiliations:** Department of Public Health, College of Medical and Health Sciences, Samara University, Semera, Afar Ethiopia

**Keywords:** Stunting, Determinants, Children, Samara University, Ethiopia

## Abstract

**Background:**

Stunting is defined as a child with a height for-age Z-score less than minus two standard deviations. Globally, 162 million less than 5 years were stunted. In Ethiopia, Nationally the prevalence of stunting among under five children was 38.4% and in Afar it is above the national average (41.1%). This study was aimed to identify determinants of stunting among children aged 6 to 59 months in rural Dubti district, Afar region, North East Ethiopia, 2017.

**Methods:**

Community based unmatched case-control study design was conducted among 322 (161 cases and 161 controls) children aged 6 to 59 months from March 2–30/ 2017. Simple random method was used to select 5 kebelles from 13 kebelles. Training was given for data collectors and supervisors. Data were entered to EPI data version 3.02 and exported to SPSS version 20 for analysis. Binary logistic regression analysis was used and variables with *p*-value < 0.25 on univariable binary logistic regression analysis were further analyzed on multivariable binary logistic regression analysis and statistical significance was declared at 95% CI.

**Results:**

Being from a mother with no education (AOR = 4.92, 95%CI (1.94, 12.4), preceding birth interval less than 24 months (AOR = 4.94, 95% (2.17, 11.2), no ANC follow-up (AOR = 2.81, 95% (1.1.46, 5.38), no access to latrine (AOR =3.26, 95% CI (1.54–6.94), children born from short mother < 150 cm (AOR = 3.75, 95%CI (1.54, 9.18), not fed colostrum (AOR = 4.45, 95% CI (1.68, 11.8), breast fed for less than 24 months (AOR = 3.14, 95% CI (1.7, 5.79) and non-exclusive breast feeding (AOR = 6.68, 95% (3.1, 14.52) were determinants of stunting at 95% CI.

**Conclusion:**

No maternal education, preceding birth interval less than 24 months, no ANC follow-up, no access to latrine, short maternal height, not feeding colostrum, duration of breast feed less than 24 months and non- exclusive breast feeding were determinants of stunting at 95% CI.

## Background

Malnutrition is a nutrient deficiency state with multiple adverse effects on human body structure and function resulting in specific physical and clinical outcomes. Stunting is one form of malnutrition (under nutrition) defined as a child with a height for-age Z-score less than minus two standard deviations [1].

About 178 million children under 5 years suffer from stunting, the vast majority in south-central Asia and sub-Saharan Africa. About 160 million (90%) live in just 36 countries, representing almost half (46%) of the 348 million children in those countries [2]. According to 2016 Ethiopian Demographic Health Survey (EDHS) key indicator report, 38.4% of Ethiopian children are stunted. Stunting prevalence in the study setting (Afar) is above the national average (41.1%) [3]. If current trends continue, projections indicate that 127 million children under 5 years will be stunted in 2025 [4].

Evidence revealed that the problems responsible for child under-nutrition are numerous and basic problems like political instability, slow economic growth and lack of education are among them. Underlying causes such as, food insecurity, lack of maternal and child care services provision, and immediate causes, like infections and inadequate dietary intake were the main factors affecting under nutrition [5].

Stunting in early life can causes increased susceptibility to infectious diseases, attenuated cognitive ability and increased behavioral problems during childhood [6]. Moreover, stunted children who experience rapid weight gain after the age of 2 years have an increased risk of becoming overweight or obese later in life. Such weight gain is also associated with higher risk of coronary heart disease, stroke, hypertension and type-2 diabetes [7].

In addition, stunting has significant educational consequences. Today in Ethiopia, more than 2 out of every 5 children are stunted. 16% of all repetitions in primary school are associated with stunting and the stunted population has on average, 1.1 years less of education [8]. According to World Bank estimates, a 1% loss in adult height due to childhood stunting is associated with a 1.4% loss in economic productivity [9]. In Ethiopia an estimated 67% of the working age population or 26 million people were stunted as children. The annual costs associated with child under nutrition are estimated at Ethiopian birr (ETB) 55.5 billion, which is equivalent to 16.5% of GDP [8]. Reduction of the prevalence to half of the current levels of child under nutrition by the year 2025 can generate annual average savings of ETB 4.4 billion (US$ 376 million) [8].

In Ethiopia, stunting prevalence decreased by 19.6% from 58% in the year 2000 to 38.4% in 2016, but the progress is still stagnant both at national and regional levels [3]. Moreover, there are studies done on determinant of stunting among children aged 6 to 59 months in different settings of Ethiopia. However, such studies are limited in pastoral community of Afar region which is lowest in infrastructure and implementation capacity than other regions. Therefore, this study was aimed to identify determinants of stunting among children aged 6 to 59 months in rural Dubti district, Afar region.

## Methods

### Study setting, design and period

Community based unmatched case control study was conducted in Dubti district from March 2–30/2017. Dubti district is located 595 km North East of Addis Ababa. It is in zone one of Afar region. Based on 2007 Ethiopian central statistical agency population projection [9], the total population and children aged 6 to 59 months were 72,906 and 2000 respectively. According to the district health office administrative report, the livelihood of the population is pastoralist and agro pastoralist. The district has 14 kebelles (the smallest administrative unit) 1 urban and 13 rural and the total households in the district were 13,071; the health service coverage was 79%. There is 1 referral hospital, 3 functional health centers, and 11 functional health posts.

### Sample size calculation

Sample size was calculated using Epi info version 7. Percent of exposure among controls and cases were 5.8 and 16.3% respectively [10]. (95% CI), 80% power, case to control ratio of 1:1, odd ratio 3.77, the sample size was153 cases and 153 controls with total sample size of 306 and considering 5% possible non-response rate. The total sample size was 322 (161 cases and 161 controls).

### Sampling procedure

Out of the total 13 rural Kebelles five rural kebelles were selected by simple random sampling technique. A house to house census was made in 5 randomly selected kebelles (the smallest administration unit in Ethiopia) to enumerate all children of age 6 to 59 months. All children aged 6 to 59 months who lived for more than 6 months in the randomly selected kebelles were enumerated. Anthropometric measurement of the children were taken for all children of age 6 to 59 months living in selected kebelles and were measured for their z-score of height for age and categorized as stunted and not stunted to generate sampling frames for cases and controls by a census conducted prior to the actual data collection. Based on this children were categorized as cases (anthropometric reading with z-scores < −2SD) or controls (anthropometric reading with z-scores ≥ −2SD) based on the median of WHO 2006 reference population. After anthropometric measurement of all the children aged 6 to 59 months was taken, children from each selected kebelle were identified and registered sequentially and got identification number and were enrolled as cases and controls. After identification of the number of cases and controls in each randomly selected kebelle, proportional allocation of samples was made in relation to the number of sample size allocated for the study. Based on this A total of 322 (161 cases and 161 controls) were taken from the randomly selected kebelles. Finally, mother -child pairs from each selected kebelle were enrolled using simple random sampling method. Interval (*K* value) was determined for each kebelle by dividing the total eligible children in the kebelle to the sample proportion. The first household was selected by lottery method. In case more than one eligible child was found in a household, only one child was selected using lottery method.

### Study variables

**Dependent variable**: Stunting.

**Independent variables:** The independent variables were socioeconomic and demographic factors (age, sex, age of mother at first birth, birth order, preceding birth interval, house hold family member, parental educational status, parental occupational status, house hold income and house hold head), environmental factors (access to toilet facility, utilization of latrine, source of water, hand washing practice and waste disposal practice), disease or morbidity factors (diarrhea, fever), feeding or dietary intake factors (time of initiating breast feed, colostrum feeding**,** duration of breastfeeding**,** method of child feeding, complementary feeding, exclusive breast feeding, pre-lacteal feeding practices, minimum dietary diversity (MDDS)), nutritional factors (size of child at birth, height of mother and body max index (BMI) of mother)) and maternal and child care factors (antenatal care visits of mother, ANC nutritional counseling, postnatal care, place of delivery and child vaccination status).

To arrive at the independent variables a review of different literatures on the subject area or similar studies conducted so far was made. UNICEF conceptual framework for causes of malnutrition (stunting) was also considered. Based on this immediate causes (inadequate intake and diseases), underline causes (household food insecurity, poor maternal and child care, lack of access to health service and unhygienic environment) and basic causes (political, ideological, economical…) causes of malnutrition were considered. Besides of this contextualization of the identified variables with livelihood of the people, with health service coverage, with health seeking behavior of the people in the pastoral community was also considered. Finally, based on the inputs from different literatures and the context in the study setting the independent variables listed above were used.

### Operational definitions of terms

The definition was taken from World Health Organization, WHO child growth standard 2006 field tables [11]. **Stunting/cases**: were defined as a children with a height for-age Z-score (HAZ) less than minus two standard deviations (<− 2 SD). **Controls:** were defined as study subjects who had anthropometric reading with z-scores ≥ − 2SD.

### Data collection tools, procedures and quality control methods

Questionnaire was initially prepared in English and Amharic and translated into the local language, Afar’af. Three days training was given for data collectors and supervisors about the data collection technique of the study. Pretested structured questionnaire, standard height measuring board and weight measurement scale was used. Calibration of weight measuring instrument was done. Pre-test was done in 5 % of the total sample in non-selected kebelles of the source population. Data were collected by 4 females trained diploma health workers with strict supervision by two trained supervisors. Mothers were interviewed about their children using pre tested questionnaire.

### Anthropometric measurements

Length of children aged 6 to 23 months was measured on recumbent position to the nearest 0.1 cm using standard length measuring board without shoes. Height of children aged 24 to 59 month was measured by placing the child in standing upright position in the middle of board wearing light clothing without shoes. The child’s head, shoulders, buttocks, knees and heels was adjusted to touch the board and each measurement was taken two times to ensure reliability of the study to the nearest 0.1 cm. Mothers who didn’t know exactly the age of their child, immunization card were used or precision in age was maintained to the nearest month. Maternal weight was measured using portable weight scale to the nearest o.1 kg and mothers were allowed not to have anything that adds to the weight being recorded. The weighting scale was checked and reset at zero point for every consecutive study subject. Maternal height was measured in standing position and measurements were made by two data collectors by holding the meter from heel to the back of head and measured to the nearest 0.1 cm.

### Data processing and analysis

Anthropometric data were calculated by using WHO Anthro2010 software and height for age Z- scores were also been generated based on the median of WHO 2006 reference population (child growth standards). Data was also entered to EpiDatav3.02 for cleaning and exported to SPSS version 20 for further analysis. Tight supervision, day to day follow up during data collection period and data cleaning before data entry were made to prevent missed data. After cleaning data for inconsistencies and missing values; descriptive statistics was done. Univariable binary logistic regression was used to assess the association of one independent variable with the dependent variable. Multivariable binary logistic regression model was used to identify potential significant determinants of stunting after control of all possible potential confounders. Variables with *p*-value < 0.25 in univariable analysis were a candidate for multivariable logistic regression analysis and statistical significance was declared at 95% CI.

Multicollinearity was checked using Variance Inflation Factor (VIF) and there was no multicollinearity (VIF < 10). Residual plots were also performed. Hosmer-Lemeshow goodness-of-fit was used to test for the model fitness and the *p*-value for Hosmer-Lemeshow test was 0.46 which indicates a good model since p-value is > 0.05 which is insignificant.

## Result

### Socio economic and demographic characteristics of study participants

A total of 322(161 cases and 161 controls) mother-child pairs were included in the study, with 100% response rate. Majority of the mothers 100 (62%) in case group and 61(38%) in control group didn’t attend any formal education. Regarding educational status of husbands, 86(54.4%) and 79 (49.7%) of husbands in case and control group didn’t attend any formal education respectively. Majority of the study participants 105(65.2%) in cases group and 104 (64.6%) in controls group were pastoralists. Around 35 and 32% of the children from the cases and controls group were aged 36 months and above respectively. Around 50.3% of the children in the cases group were females and 54.7% of the children in the controls group were males. Birth interval (≥ 24 months) was 57.8 and 71.4% in the cases and controls group respectively (Table 1).

**Table 1:** Socio demographic and economic characteristics of children aged 6 to 59 months in rural Dubti district, Afar region North East Ethiopia, 2017: *n* = 322

Variable(s)	Cases (%)	Control (%)	Total (%)
Maternal education			
No education	100 (62)	61 (38)	161 (50)
Primary	32 (19.9)	44 (27.3)	76 (23.6)
Secondary	12 (7.5)	23 (14.3)	35 (10.9)
Higher	17 (10.6)	33 (20.5)	50 (15.5)
Father educational status			
No education	86 (54.4)	79 (49.7)	165 (52.1)
Primary	29 (18.4)	27 (17)	56 (17.7)
Secondary	34 (21.5)	30 (18.9)	64 (20.2)
Higher	9 (5.7)	23 (14.5)	32 (10)
Maternal occupation			
Pastoral	105 (65.2)	104 (64.6)	209 (65)
Government	39 (24.2)	45 (28)	84 (26)
Other	17 (10.6)	12 (7.4)	29 (9)
Age of the child			
6–11	20 (12.4)	31 (19.3)	51 (15.8)
12–23	37 (23)	36 (22.4)	73 (22.7)
24–35	48 (29.8)	43 (26.7)	91 (28.3)
36 and above	56 (34.8)	51 (31.7)	107 (33.2)
Sex			
Male	80 (49.7)	88 (54.7)	168 (52.2)
Female	81 (50.3)	73 (45.3)	154 (47.8)
Birth order			
1	32 (19.9)	34 (21.1)	66 (20.5)
2–3	79 (49.1)	83 (51.6)	162 (50.3)
≥ 4	50 (31.1)	44 (27.3)	94 (29.2)
Preceding birth interval			
No previous birth	25 (15.5)	28 (17.4)	53 (16.5)
< 24 months	43 (26.7)	18 (11.2)	61 (18.9)
≥ 24 months	93 (57.8)	115 (71.4)	208 (64.6)
Household family member			
2–4	83 (51.6)	88 (54.7)	171 (53)
≥ 5	78 (48.4)	73 (45.3)	151 (47)

### Maternal and child care characteristics of study participants

Thirty eight (23.6%) of mothers of children in cases group and fifty (31.1%) mothers of children in controls group had ANC follow up. Regarding delivery, One hundred twenty two (75.8%) and 111 (68.9%) mothers in cases and controls group had home delivery respectively. Around 8 and 9% mothers from cases group and controls group attend post natal care visit respectively (Table 2).

**Table 2:** Maternal and child care characteristics of children aged 6 to 59 months in rural Dubti district, Afar region North East Ethiopia, 2017: *n* = 322

Variable(s)	Case (%)	Control (%)	Total (%)
ANC follow-up			
Yes	38 (23.6)	50 (31.1)	88 (27.3)
No	123 (76.4)	111 (68.9)	234 (72.7)
Number of ANC visit			
1	13 (34.2)	11 (22)	24 (27.3)
2–3	15 (39.5)	23 (46)	38 (43.2)
≥ 4	10 (26.3)	16 (32)	26 (29.5)
Place of delivery			
Home	122 (75.8)	111 (68.9)	233 (72.4)
Facility	39 (24.2)	50 (31.1)	89 (27.6)
Post natal care visit			
Yes	13 (8.1)	15 (9.3)	28 (8.7)
No	148 (91.9)	146 (90.7)	294 (91.3)
Maternal BMI			
18.5–24.9 kg/m^2^	88 (54.7)	84 (52.2)	172 (53.4)
< 18.5 kg/m^2^	46 (28.6)	31 (19.3)	77 (23.9)
≥ 25 kg/m^2^	27 (16.8)	46 (28.6)	73 (22.7)

### Child feeding and environmental characteristics of study subjects

One hundred six (65.5%) of children from the cases group and 215(66.8%) of children from the controls group were initiated breast feeding immediately after birth. One hundred thirty five (83.9%) of cases and 151(93.8%) controls were feed colostrum. Prelacteal feeding was practiced in 44.7 and 29.2% of the children in cases and controls group respectively. Exclusive breastfeeding was practiced in 53.4 and 67.7% of cases and controls respectively. Around 51.6% of children in the cases group and 76.4% of children in the controls group continued breastfeeding till 2 years of age. Of the total participants 42(26.1%) of cases and 77(47.8%) controls had latrine. Of those who had latrine, 73.8 and 76.6% of participants from the cases group and control group had functional latrine respectively. Two hundred forty (74.5%) of household study subjects had protected water supply. Regarding source of drinking water, 47(29.2%) of cases and 35(21.7%) of controls had unprotected water supply (Table 3).

**Table 3:** Child feeding and Environmental characteristics of children aged 6 to 59 months in rural Dubti district, Afar region North East Ethiopia, 2017: *n* = 322

Variable(s)	Case (%)	Control (%)	Total (%)
Colostrum feeding			
Yes	135 (83.9)	151 (93.8)	286 (88.8)
No	26 (16.1)	10 (6.2)	36 (11.2)
Prelacteal feeding			
Yes	72 (44.7)	47 (29.2)	119 (37)
No	89 (55.3)	114 (70.8)	203 (63)
Duration of breastfeeding			
< 24	78 (48.4)	38 (23.6)	116 (36)
≥ 24 months	83 (51.6)	123 (76.4)	206 (64)
Duration of exclusive breast feeding			
Less 6 months	75 (46.6)	52 (32.3)	127 (39.4)
At 6 month	86 (53.4)	109 (67.7)	195 (60.6)
Age Complementary feeding started			
Less than 6 months	75 (46.6)	52 (32.3)	127 (39.4)
At 6 months	86 (53.4)	109 (67.7)	195 (60.6)
Availability of latrine			
Yes	42 (26.1)	77 (47.8)	119 (37)
No	119 (73.9)	84 (52.2)	203 (63)
Functional latrine			
Yes	31 (73.8)	59 (76.6)	90 (75.6)
No	11 (26.2)	18 (23.4)	29 (24.4)
Source of drinking water			
Unprotected source	47 (29.2)	35 (21.7)	82 (25.5)
Protected source	114 (70.8)	126 (78.3)	240 (74.5)

### Factors associated with stunting

Based on multivariable binary logistic regression analysis, Being from a mother with no education (AOR = 4.92, 95%CI (1.94, 12.4), preceding birth interval less than 24 months (AOR = 4.94, 95% (2.17, 11.2), no ANC follow-up (AOR = 2.81, 95% (1.1.46, 5.38), no access to latrine (AOR =3.26, 95% CI (1.54–6.94), children born from short mother < 150 cm (AOR = 3.75, 95%CI (1.54, 9.18), not fed colostrum (AOR = 4.45, 95% CI (1.68, 11.8), breast fed for less than 24 months (AOR = 3.14, 95% CI (1.7, 5.79) and non-exclusive breast feeding (AOR = 6.68, 95% (3.1, 14.52) were determinants of stunting at 95% CI. Having this, Children born to mothers with no education were 4.9 times more likely to be stunted comparing to children born to mothers with higher education (AOR = 4.92, 95%CI (1.94, 12.4). Children born with birth interval of < 2 years were 4.9 times more likely to be stunted comparing to children born with birth interval of ≥2 years (AOR = 4.94, 95% (2.17, 11.2). children born to a mother with no ANC follow-up were 2.8 times more likely to be stunted comparing to their counterparts (AOR = 2.81, 95% (1.1.46, 5.38) and children born to a household with no access to latrine were 3 times more likely to be stunted comparing to their counterparts (AOR =3.26, 95% CI (1.54–6.94). Children born to short mothers (height < 150 cm) were 3.7 times more likely to be stunted comparing to their counterparts (AOR = 3.75, 95%CI (1.54, 9.18), children whose mothers’ squeezed out colostrums twofold times more likely contributes to stunting than who fed their children colostrums (AOR = 4.45, 95% CI (1.68, 11.8), and children who had breastfeeding for less than 24 months were threefold times more likely to be stunted than who continued breastfeeding till 2 years and above (AOR = 3.14, 95% CI (1.7, 5.79). Non-exclusive breast-fed children were 6.6 times more likely to be stunted comparing to their counterparts (AOR = 6.68, 95% (3.1, 14.52) (Table 4).

**Table 4:** Binary logistic regression analysis showing determinant of stunting in rural Dubti district, Afar region, North East Ethiopia 2017; *n* = 322

Variable(s)	Case (%)	Control (%)	COR(95% CI)	AOR((95% CI)
Maternal education				
No education	100 (62.1)	61 (37.9)	3.18 (1.64, 6.19)	4.92 (1.94, 12.4)*
Primary	32 (19.9)	44 (27.3)	1.41 (0.67, 2.96)	2.21 (0.87, 5.61)
Secondary	12 (7.5)	23 (14.3)	1.01 (0.41, 2.52)	0.66 (0.22, 2.01)
Higher	17 (10.6)	33 (20.5)	1	1
Preceding birth interval				
No previous birth	25 (15.5)	28 (17.4)	1.1 (0.6–2.2)	1.1 (0.51, 2.35)
< 24 months	43 (26.7)	18 (11.2)	2.95 (1.59–5.54)*	4.94 (2.17, 11.2)*
≥ 24 months	93 (57.8)	115 (71.4)	1	1
ANC follow-up				
Yes	38 (23.6)	50 (31.1)	1	1
No	123 (76.4)	111 (68.9)	1.46 (0.89–2.39)	2.81 (1.46, 5.38)*
Place of delivery				
Home	122 (75.8)	111 (68.9)	1.41 (0.86–2.3)	1.74 (0.91, 3.3)
Facility	39 (24.2)	50 (31.1)	1	1
House hold water supply				
Un protected	47 (29.2)	35 (21.7)	1.48 (0.89–2.46)	0.85 (0.44, 1.66)
Protected	114 (70.8)	126 (78.3)	1	1
Latrine status				
Yes	42 (26.1)	77 (47.8)	1	1
No	119 (73.9)	84 (52.2)	2.59 (1.63–4.15)*	3.26 (1.54–6.94)*
Maternal height				
< 150 cm	27 (16.8)	13 (8.1)	3.4 (1.62–7.27)*	3.75 (1.54, 9.18)*
15–154.9 cm	46 (28.6)	22 (13.7)	3.45 (1.86–6.42)*	3.48 (1.63–7.45)*
155–159.9 cm	39 (24.2)	45 (28)	1.43 (0.82–2.5)	1.06 (0.52, 2.2)
> =160 cm	49 (30.4)	81 (50.3)	1	1
Colostrum feeding				
Yes	135 (83.9)	151 (93.8)	1	1
No	26 (16.1)	10 (6.2)	2.91 (1.35–6.25)*	4.45 (1.68, 11.8)*
Pre lacteal feeding				
Yes	72 (44.7)	47 (29.2)	1.96 (1.24–3.12)*	1.35 (0.74, 2.5)
No	89 (55.3)	114 (70.8)	1	1
Duration of breast feeding				
< 24	78 (48.4)	38 (23.6)	3.04 (1.88–4.9)*	3.14 (1.7, 5.79)*
≥ 24 months	83 (51.6)	123 (76.4)	1	1
Duration of exclusive breast feeding				
Less 6 months	75 (46.6)	52 (32.3)	1.82 (1.16–2.87)*	6.68 (3.1, 14.52)*
> =6 months	86 (53.4)	109 (67.7)	1	1

*COR* Crude odds ratio, *AOR* Adjusted odds ratio, *CI* confidence interval

*significant at *p* < 0.05

## Discussion

In this study, No maternal education, preceding birth interval less than 24 months, no ANC follow-up, no access to latrine, short maternal height, not feeding colostrum, duration of breastfeeding less than 24 months and non-exclusive breast feeding were determinants of stunting at 95% CI. Children born to mothers with no education were 4.9 times more likely to be stunted comparing to children born to mothers with higher education (AOR = 4.92, 95%CI (1.94, 12.4). This is in line with the study conducted in Tanzania, Malawi, and Nigeria [12–14]. This might be educated mothers have better health-seeking behavior for childhood illnesses as compared to uneducated mothers which can help prevent stunting [15].

Children born to a household with no access to latrine were 3 times more likely to be stunted comparing to their counterparts (AOR =3.26, 95% CI (1.54–6.94). This is consistence with the study done in Ethiopia [16]. This might be due to a reduction in the pathogen load in the environment from correct and consistent use of improved sanitation [17]. Children whose mothers’ squeezed out colostrums twofold times more likely contributes to stunting than who fed their children colostrums (AOR = 4.45, 95% CI (1.68, 11.8). This is consistence with finding in west Gojjam [18]. This might be probably colostrum provides protective effect to the newborns. Children born with birth interval of < 2 years were 4.9 times more likely to be stunted comparing to children born with birth interval of ≥2 years (AOR = 4.94, 95% (2.17, 11.2). This is consistence with the study conducted in Gurage zone [10]. This might be continued breastfeeding in the second year contributes significantly intake of key nutrients that are lacking in low-quality complementary diets [19]. This was also in line with the study done in Ethiopia and Cambodia [20, 21]. This might be explained by; a short birth interval between births can have an adverse effect on child nutrition by causing intrauterine growth retardation, and/or undermining the quality of child care [22].

Non-exclusive breast-fed children were 6.6 times more likely to be stunted comparing to their counterparts (AOR = 6.68, 95% (3.1, 14.52). This finding was consistence with the study conducted in Gurage zone and Bangladesh [10, 23]. This might be early initiation and exclusive breastfeeding for 6 months provides protection against gastrointestinal infections, which can lead to severe nutrient depletion and stunting [24]. Children born to short mothers (height < 150 cm) were 3.7 times more likely to be stunted comparing to their counterparts (AOR = 3.75, 95%CI (1.54, 9.18). This was consistence with the study [25]. This might be Short maternal stature is associated with intrauterine growth retardation and low birth weight, which are in turn determinants of infant death and impaired child growth [26].

The odd of no ANC follow up was 2.81 time higher among cases of mothers than those that had ANC follow up (AOR = 2.81, 95% (1.1.46, 5.38). Similar study was reported at Afambo district and Tanzanian [27, 28]. This study reported that, lack of antenatal visit might create favorable environment for traditional child feeding malpractices that might affect child nutritional status.

The study might have the following limitation, recall bias, since it is a relatively long period to expect people to remember, but the recall problem hopefully was of no differential nature. There might be also misclassification of case and control, because it is very difficult to get accurate age particularly in rural community. However, due attention was given to the study procedures, including the process of training, standardization of anthropometric measurements, and close supervision throughout the field activities. Since case-control study design was employed, it does not enable to establish temporality.

## Conclusion

This study has shown that the independent predicators for stunting were no formal maternal education, no access to latrine, short maternal height, duration of breast feed less than 24 month, exclusively breast feeding before 6 month, complimentary feeding before 6 month, not feeding colostrum, not attending ANC follow up and preceding birth interval less than 24 months. Therefore, the regional health bureau and Dubti district health office should increase awareness creation to bring behavioral change at community level on exclusive breast feeding, access to latrine maternal education, colostrum feeding, birth spacing for at least > 24 month, duration of breast feeding for 24 or more months, complementary feeding at 6 month and ANC follow up.

## Data Availability

The datasets supporting the conclusions of the study are included in the article. Any additional data will be available on request. The datasets used and/or analysed during the current study are available from the corresponding author on reasonable request.

## Abbreviations

AOR: Adjusted Odds Ratio
BMI: Body Mass Index
CI: Confidence Interval
COR: Crude Odds Ratio
EDHS: Ethiopian Demographic and Health Survey
MDDS: Maternal Dietary Diversity Score
SPSS: Statistical Package for Social Science
WHO: World Health Organization
